# Supplementary material for: Antibiofilm Activities of Multiple Halogenated Pyrimidines Against Staphylococcus aureus
Source: Int J Mol Sci. 2024 Nov 28;25(23):12830. doi: 10.3390/ijms252312830 (PMC11641129; doi:10.3390/ijms252312830)
Supplement: Supplementary file 1 [file ijms-25-12830-s001.zip › ijms-3319365-supplementary.pdf]

International Journal of
Molecular Sciences

MDPI

Article

Antibiofilm activities of multiple halogenated pyrimidines against *Staphylococcus aureus*

MinHwi Sim [1], Yong-Guy Kim [1], Jin-Hyung Lee [1,*] and Jintae Lee [1,*]

[1] School of Chemical Engineering, Yeungnam University, Gyeongsan, 38541, Republic of Korea; tla7686@yu.ac.kr (M.S.); yongguy7@ynu.ac.kr (Y.-G.K.)

* Correspondence: jinhlee@ynu.ac.kr (J.-H.L.); jtlee@ynu.ac.kr (J.L.); Tel.: +82-53-810-3812 (J.-H.L.); Tel.: +82-53-810-2533 (J.L.)

1. Supplementary

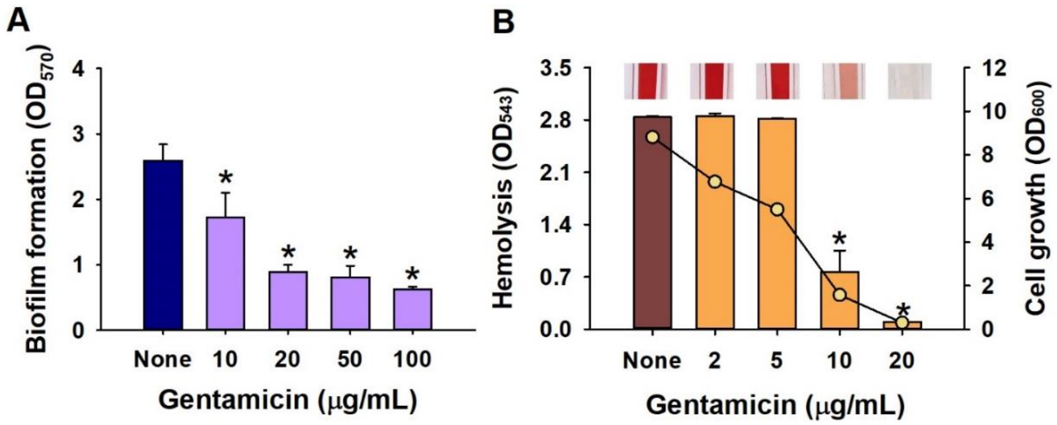

Figure S1. Dose-dependent inhibition of biofilm formation by gentamicin (A). Hemolytic activity at different gentamicin concentrations (B). *$p < 0.05$ *vs.* untreated controls (None).

Citation: Sim, M.; Kim, Y.-G.; Lee, J.-H.; Lee, J. Antibiofilm activities of multiple halogenated pyrimidines against *Staphylococcus aureus*. *Int. J. Mol. Sci.* **2024**, *25*, x. https://doi.org/10.3390/xxxxx

Academic Editor(s): Name

Received: date
Revised: date
Accepted: date
Published: date

Copyright: © 2024 by the authors. Submitted for possible open access publication under the terms and conditions of the Creative Commons Attribution (CC BY) license (https://creativecommons.org/licenses/by/4.0/).
